# Ubiquitously expressed transcript is a novel interacting protein of protein inhibitor of activated signal transducer and activator of transcription 2

**DOI:** 10.3892/mmr.2014.3023

**Published:** 2014-12-01

**Authors:** XIANG KONG, SHIKUN MA, JIAQIAN GUO, YAN MA, YANQIU HU, JIANJUN WANG, YING ZHENG

**Affiliations:** 1 Department of Gynecology and Obstetrics, Medical College of Yangzhou University, Yangzhou, Jiangsu 225001, P.R. China; 2 Department of Histology and Embryology, Medical College of Yangzhou University, Yangzhou, Jiangsu 225001, P.R. China; 3 Reproductive Medicine Center, Medical College of Yangzhou University, Yangzhou, Jiangsu 225001, P.R. China

**Keywords:** yeast two-hybrid system, PIAS2, UXT, protein interaction

## Abstract

Protein inhibitor of activated signal transducer and activator of transcription 2 (PIAS2) is a member of the PIAS protein family. This protein family modulates the activity of several transcription factors and acts as an E3 ubiquitin ligase in the sumoylation pathway. To improve understanding of the physiological roles of PIAS2, the current study used a yeast two-hybrid system to screen mouse stem cell cDNA libraries for proteins that interact with PIAS2. The screening identified an interaction between PIAS2 and ubiquitously expressed transcript (UXT). UXT, also termed androgen receptor trapped clone-27, is an α-class prefoldin-type chaperone that acts as a coregulator for various transcription factors, including nuclear factor-κB and androgen receptor (AR). A direct interaction between PIAS2 and UXT was confirmed by direct yeast two-hybrid analysis. *In vitro* evidence of the association of UXT with PIAS2 was obtained by co-immunoprecipitation. Colocalization between PIAS2 and UXT was identified in the nucleus and cytoplasm of HEK 293T and human cervical carcinoma HeLa cells. The results of the current study suggested that UXT is a binding protein of PIAS2, and interaction between PIAS2 and UXT may be important for the transcriptional activation of AR.

## Introduction

Ubiquitously expressed transcript (UXT), located on the Xp11.23-p11.22 chromosome, is a widely expressed gene in humans and mice and is upregulated in certain tumors (1). UXT has two isoforms: UXT-V2, the short form that consists of 157 amino acids and is primarily expressed in the nucleus; and UXT-V1, which is 169 amino acids in length and is predominantly expressed in the cytoplasm (2,3). UXT interacts with the N-terminus of the androgen receptor (AR) and regulates androgen-responsive genes (3,4); it has also been described as a suppressor of cell transformation and a coregulator of nuclear factor-κB (5,6). High expression of UXT has been demonstrated to result in mitochondrial aggregation (7), and one study identified that UXT-V1 protects the cells from TNF-induced apoptosis (8). Under conditions of infection and inflammation, UXT has dual opposing effects on SARM (sterile α and HEAT/armadillo motif protein)-induced apoptosis. UXT-V2 transfection previously resulted in cell death and a reduced mitochondrial membrane potential when cotransfected with SARM (9). By contrast, UXT-V1 cotransfected with SARM has been demonstrated to led to a significant reduction in caspase 8 activity (9).

Protein inhibitor of activated signal transducer and activator of transcription 2 (PIAS2) is a member of the PIAS protein family (10,11). In mammals, four members of the PIAS protein family have been identified: PIAS1 (12), PIAS2 (13), PIAS3 (14) and PIAS4 (15). PIAS proteins were previously thought to be inhibitors of activated STAT only (16), but are now known to interact with and modulate several other proteins, including AR and p53 (13,17). In addition, PIAS proteins act as E3 ligases in sumoylation, but appear to possess functions beyond the modification process of sumoylation (18,19). The PIAS2 gene encodes two splice variants, PIASxα/androgen receptor-interacting protein-3 (ARIP3) and PIASxβ, which have different C termini (13,20). PIAS2 is highly expressed in the testis and its protein is detected until stage XII of the seminiferous epithelium in spermatogonia and pachytene spermatocytes, in addition to Sertoli cells, suggesting a role for PIAS2 in testicular function (21).

The functional regulators of PIAS2 in spermatogenesis remain largely unknown, thus, in the current study, cDNAs encoding PIAS2-binding proteins were screened using the yeast two-hybrid system. UXT was identified as a novel PIAS2-binding protein and was suggested to physically interact with PIAS2.

## Materials and methods

### Reagents and antibodies

Mouse anti-human monoclonal anti-c-Myc Tag antibody, was purchased from EMD Millipore (cat no. 4A6, 05–724; Billerica, MA, USA). Rabbit anti-green fluorescent protein (GFP) polyclonal antibody was purchased from Epitomics (cat no. ab137828; Burlingame, CA, USA).

### Construction of plasmids

The cDNA fragment of the mouse PIAS2 gene (9–401 aa corresponding to nucleotides 213–1391 of NM_008602) was cloned into a pGBKT7 vector (cat no. 630489; Clontech Laboratories, Inc., Mountainview, CA, USA) containing the GAL4 DNA-binding domain. This generated the bait plasmid, pGBKT7-PIAS2. This construct was not observed to produce toxic effects or autonomous transcriptional activation following transformation into the yeast strain, Y187. This plasmid was used for the yeast two-hybrid screening.

Polymerase chain reaction (PCR) was used to amplify the common cDNA fragment of the mouse PIAS2 gene, which was then cloned into the pCMV-c-Myc (cat no. 631604; Clontech Laboratories, Inc.) and pDsRed-Express-1 vectors (cat no. 632413; Clontech Laboratories, Inc.) to generate pCMV-c-Myc-PIAS2 and pDsRed-Express-1-PIAS2, respectively. Full-length UXT was cloned into a pEGFP-N1 vector (cat no. 6085-1; Clontech, Laboratories, Inc.) to generate pEGFP-N1-UXT. pCMV-c-Myc-PIAS2 and pEGFP-N1-UXT were used in the Co-IP assay, while pDsRed-Express-1-PIAS2 and pEGFP-N1-UXT were used in the colocalization analysis.

### Yeast two-hybrid system

cDNA libraries for the mouse stem cells were constructed in a pGADT7-Rec vector containing a GAL4 activation domain using Matchmaker Library Construction and Screening Kits (cat no. 630445; Clontech Laboratories, Inc.) and then transformed into the yeast AH109 strain. Yeast two-hybrid screening was conducted using the Matchmaker Gold Yeast Two-Hybrid System (cat no. 630489; Clontech Laboratories, Inc.). Positive clones were selected based on ability to grow on synthetic dropout medium (cat no. 630412; Clontech Laboratories, Inc.) with an absence of leucine, tryptophan, histidine and adenine (SD/-LTHA)/X-α-Gal (cat no. 630407; Clontech Laboratories, Inc.) and for α-galactosidase activity.

In the Matchmaker Two-Hybrid assay, pGBKT7 expresses proteins as fusions to the GAL4 DNA-BD, while pGADT7-Rec expresses proteins as fusions to the GAL4 AD. A bait gene is expressed as a fusion to the GAL4 DNA-binding domain (DNA-BD), while another gene or cDNA is expressed as a fusion to the GAL4 activation domain (AD). When bait (recombined pGBKT7) and library fusion proteins interact in a yeast reporter strain such as AH109, the DNA-BD and AD are brought into proximity and activate transcription of the reporter genes (ADE2, HIS3, lacZ, and MEL1). It is known that P53 can interact with Simian virus 40 T large antigen (SV40), thus, these were used as a positive control. When cotransformed pGBKT7-p53 and pGADT7-SV40 into AH109, they can interact and activate transcription of and thus induce expression of the reporter genes (ADE2, HIS3, lacZ, and MEL1). Thus, the transformed AH109 strain can grow on an SD/-LTHA plate. X-α-Gal is a chromogenic substrate for α-galactosidase. In the AH109 strain, this enzyme is encoded by the MEL1 gene, which is regulated by several GAL genes. Secretion of this enzyme in response to GAL4 activation leads to hydrolysis of X-α-Gal in the medium, resulting in yeast colonies developing a blue color.

Plasmid DNA of prey clones was isolated using QIAprep Spin Miniprep kit according to the manufacturer’s instructions (cat no. 27104; Qiagen, Hilden, Germany) and transformed into *E. coli* DH5α (cat no. D9057; Takara Bio Inc. Otsu, Japan). Subsequently, prey clones were recovered in the lysogeny broth medium (Thermo Fisher Scientific, Inc., Waltham, MA, USA) containing ampicillin. and cDNA inserts were amplified by PCR using the Advantage 2 PCR kit (cat no. 639207; Clontech Laboratories, Inc.). The primer sequences used for PCR were as follows: Forward: 5′-TTCCACCCAAGCAGTGGTATCAACGCAGAGTGG-3′ and Reverse: 5′-GTATCGATGCCCACCCTCTAGAGGCCGAGGCGGCCGACA-3′. PCR was performed at 94°C for 3 min; 30 cycles of 94°C for 30 sec and 68°C for 3 min; 68°C for 3 min; and then maintained at 15°C. The PCR products were sequenced and then analyzed using the basic local alignment search tool (BLAST; http://blast.ncbi.nlm.nih.gov/Blast.cgi). Interaction between the bait and identified prey clones was verified by cotransforming the purified prey plasmid with the bait pGBKT7-PIAS2 construct into the yeast AH109 strain, followed by selection on SD/-LTHA medium. Cotransformation of pGBKT7-p53 with pGADT7-SV40 was used as a positive control. A negative control was also produced by the cotransformation of pGBKT7-UXT with the pGADT7 vector into the AH109 yeast cells.

### Mammalian cell culture and transient transfection assay

A human cervical carcinoma (HeLa) and SV40 T-antigen-expressing human embryonic kidney (HEK 293T) cell lines were cultured and maintained in Dulbecco’s modified Eagle’s medium (DMEM; Invitrogen, Life Technologies, Carlsbad, CA, USA) supplemented with 10% fetal bovine serum (FBS; GE Healthcare Life Sciences, Logan, UT, USA) and 50 U/ml each of penicillin and streptomycin (Invitrogen Life Technologies), at 37°C in a humidified atmosphere with 5% CO_2_. For the transient transfections, HEK 293T and HeLa cells were seeded into 6-well plates at 70~90% confluence, washed once with DMEM and transfected with 2 μg pCMV-c-Myc-PIAS2 and 2 μg pEGFP-N1-UXT (or 2 μg pDsRed-Express-1-PIAS2 and 2 μg pEGFP-N1-UXT) using 12 μl Lipofectamine 2000 reagent (Invitrogen Life Technologies) in a total of 1 ml serum-free DMEM per 25-mm well, in accordance with the manufacturer’s instructions. Approximately 3 h following transfection, the transfection mixture was replaced with 2 ml DMEM-10% FBS, rested for 3–5 h and then replaced again with fresh DMEM-10% FBS.

### Co-IP

The HEK 293T cells were transiently transfected with pCMV-c-Myc-PIAS2 and pEGFP-N1-UXT constructs, then were maintained as described above for 48 h. The cells were then washed with ice-cold phosphate-buffered saline (PBS; cat no. ZLI-9061; Origene, Beijing, China) and harvested in 500 ml lysis buffer [150 mM NaCl; 50 mM Tris, pH 8.0; 1% Nonidet P-40; 0.5% deoxycholate; and a protease inhibitor mixture (Roche Diagnostics GmbH, Mannheim, Germany)]. Subsequent to 60-min incubation in lysis buffer on ice, lysates were clarified by centrifugation for 20 min at 16,873 × g at 4°C. Anti-Myc or anti-GFP antibody (8 μg) was added to the supernatant and incubated for 120 min at 4°C prior to the addition of 50 μl protein G-agarose (cat no. 11719386001; Hoffmann-La Roche Ltd., Basel, Switzerland) and incubation at 4°C overnight. Samples were then centrifuged for 1 min in a microcentrifuge at 16,873 × g (5424; Eppendorf, Hamburg, Germany) and washed with 1 ml lysis buffer, 1 ml washing buffer (500 mM NaCl; 50 mM Tris, pH 7.5; 0.1% Nonidet P-40; 0.05% deoxycholate) and 1 ml washing buffer (10 mM Tris, pH 8.0; 0.1% Nonidet P-40; 0.05% deoxycholate).

### Western blotting

Initial lysates and immunoprecipitated proteins were analyzed using SDS-PAGE. SDS buffer and 12% polyacrylamide gels were prepared with reagents purchased from Invitrogen Life Technologies. Electrophoresis was carried out using the Mini-PROTEAN Tetra Cell-2 gel system (Bio-Rad Laboratories, Inc., Hercules, CA, USA). Immunoblotting was carried out using anti-GFP or anti-Myc antibody. Proteins were transferred to nitrocellulose membranes (GE Healthcare Life Sciences, Chalfont, UK) by electrophoresis and were probed with the appropriate antibodies. Anti-GFP or anti-Myc antibody was visualized with horseradish peroxidase (HRP)-conjugated anti-rabbit (cat no. A9169; Sigma-Aldrich, St. Louis, MO, USA) or HRP conjugated goat anti mouse IgG antibodies (cat no. A9044; Sigma-Aldrich) using the Pierce ECL Plus western blotting substrate (Thermo Fisher Scientific, Inc.).

### Fluoresence microscopy

To detect PIAS2 and UXT, HEK 293T and HeLa cells plated on round coverslips were transiently transfected with the pDsRed-Express-1-PIAS2 and pEGFP-N1-UXT constructs using Lipofectamine 2000. Cells were washed twice with PBS 48 h later and visualized using a scanning laser confocal microscope (Nikon A1; Nikon Corporation, Tokyo, Japan).

## Results

### UXT interacts with PIAS2

**Figure 1:**
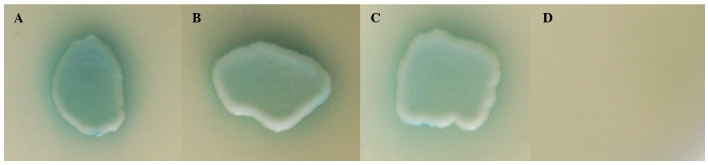
Direct yeast two-hybrid analysis, used to confirm the interaction between UXT and PIAS2. (A and B) UXT prey cotransformed with the pGBKT7-PIAS2; (C) positive control (pGBKT7-p53 and pGADT7-Simian virus 40 T large antigen) and (D) negative control (pGADT7-UXT and pGBKT7). UXT, ubiquitously expressed transcript; PIAS2, protein inhibitor of activated signal transducer and activator of transcription 2.

To further understand the function of PIAS2, the common cDNA fragment of the mouse PIAS2 gene was used as an interaction trap for interactive substrates expressed by a mouse stem cell cDNA library with the Matchmaker Library Construction and Screening kit. Several positive clones that demonstrated strong growth on the SD/-LTHA medium were isolated, sequenced and aligned using the NCBI BLAST. Among these positive clones, two clones that encoded the same region of UXT, spanning a region of 147 amino acids (UXT residues 11–157), were observed to interact with the PIAS2 protein. The interaction specificity between PIAS2 and UXT was confirmed by the direct yeast two-hybrid assay. Strong growth on SD/-LTHA medium was observed in yeast cells cotransformed with pGBKT7-PIAS2 and pGADT7-UXT (Fig. 1A and B), indicating an interaction between UXT and PIAS2 in yeast. Several positive clones that demonstrated strong growth on the SD/-LTHA medium were isolated, sequenced and aligned using the NCBI BLAST. Among these positive clones, two clones that encoded the same region of UXT, spanning a region of 147 amino acids (UXT residues 11–157), were observed to interact with the PIAS2 protein. Fig. 1A and B exhibit two different cDNA clones screened from the cDNA library. Growth was also observed in the positive control (Fig. 1C). However, the yeast strain cotransformed with pGADT7-UXT and pGBKT7 did not grow on SD/-LTHA medium and exhibited negative α-galactosidase activity (Fig. 1D).

### UXT interacts with PIAS2 in mammalian cells

**Figure 2:**
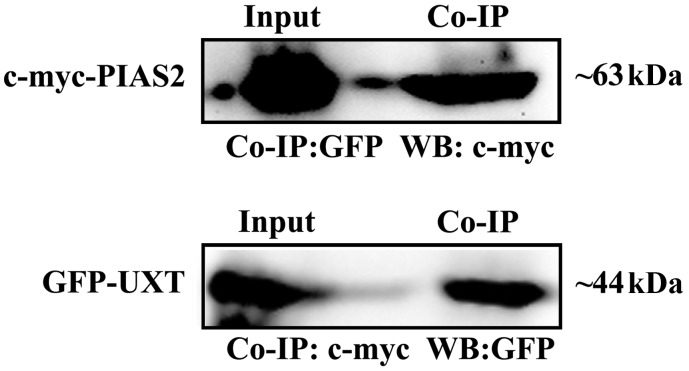
UXT interaction with PIAS2 in mammalian cells. Co-IP analysis by WB, in addition to analysis of the total lysates (Input). UXT, ubiquitously expressed transcript; PIAS2, protein inhibitor of activated signal transducer and activator of transcription 2; Co-IP, co-immunoprecipitation; WB, western blotting; GFP, green fluorescent protein.

In order to confirm the results of the two-hybrid screen using an independent biochemical method, it was determined whether PIAS2 and UXT proteins bind tightly enough to each other to be co-immunoprecipitated. The expression vectors for c-myc-tagged PIAS2 and GFP-tagged UXT were cotransfected into HEK 293T cells. The cell extract was prepared and the proteins in the extract were immunoprecipitated with anti-c-myc and anti-GFP, respectively, 24 h subsequent to transfection. The precipitates were then immunoblotted against the anti-GFP or anti-c-myc antibodies. The anti-c-myc antibody precipitated c-myc-PIAS2, whereas GFP-UXT was detected in the immunoprecipitate with the anti-GFP antibody (Fig. 2). PIAS2 protein was also observed to be immunoprecipitated from the transfected HEK 293T cells, using a rabbit anti-GFP antibody prior to detection of PIAS2 by the anti-c-myc antibody. As demonstrated in Fig. 2, UXT and PIAS2 fusion proteins were observed in the Co-IP and total lysate (Input). These results indicate that UXT may interact with PIAS2 in mammalian cells.

### UXT colocalized with PIAS2 in HEK 293T and HeLa cells

**Figure 3:**
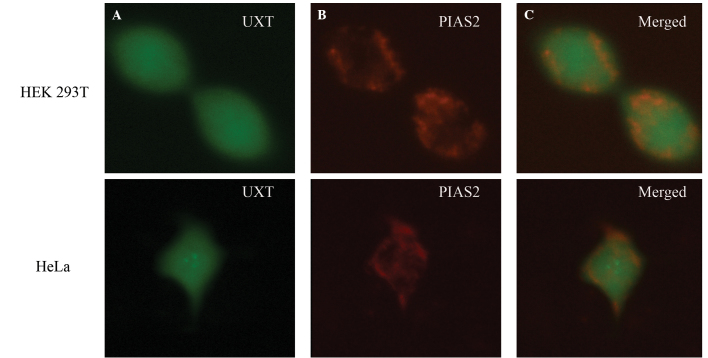
UXT colocalization with PIAS2 in HEK 293T and HeLa cells, observed using fluorescence microscopy. (A) Cell transfection with pEGFP-N1-UXT or (B) DsRed-PIAS2. (C) Merged image, exhibiting the colocalization of UXT and PIAS2. UXT, ubiquitously expressed transcript; PIAS2, protein inhibitor of activated signal transducer and activator of transcription 2.

The interaction between the PIAS2 and UXT proteins was further determined by colocalization analysis. The pDsRed-Express-1-PIAS2 and pEGFP-N1-UXT plasmids were cotransfected into HEK 293T and HeLa cells, respectively, then the cells were detected using a scanning laser confocal microscope two days subsequent to transfection. As demonstrated in Fig. 3, GFP-UXT protein was distributed in the cytoplasm and nucleus of HEK 293T and HeLa cells (Fig. 3A), as was DsRed-PIAS2 protein (Fig. 3B). Fig. 3C indicates that PIAS2 and UXT were partially colocalized in these cells, implicating a potential interaction between UXT and PIAS2 in HEK 293T and HeLa cells.

## Discussion

The aim of the current study was to elucidate the protein interactions of PIAS2. There are two isoforms of human PIAS2, including PIASxα and PIASxβ, whereas there are five isoforms of the mouse PIAS2 gene (isoforms 1–5), which have different N- and C-termini as a result of alternative splicing. Thus, the common encoding region of mouse PIAS2 as a bait to screen a mouse cDNA library. UXT protein, which coregulates various transcription factors, including AR, was observed to specifically interact with PIAS2 *in vitro*. In addition, PIAS2 and UXT were observed to colocalize in the nucleus and cytoplasm, suggesting that they have the ability to synchronously interact with each other in mammalian cells.

The AR, a member of the steroid receptor superfamily, is an X-linked nuclear receptor (NR) that is regarded as critical in sexual differentiation, gonadal maturation, maintenance of secondary male characteristics and the development of prostate cancer (3,4). Upon binding to androgen, the AR is released from heat-shock proteins, forms homodimers and is translocated into the nucleus. The AR is a single polypeptide with four functional domains, including the NH2-terminal transactivation domain; the DNA-binding domain (DBD); the hinge region; and the ligand-binding domain (LBD) (22). The transcriptional activation functions (AFs) of AR represent surfaces that are able to interact with transcription factors and additional coactivators. Coactivators that interact with the AR N-terminal AF-1 and the C-terminal AF-2 region, which leads to the enhancement of AR-dependent gene transcription, have been identified (23,24). Regions of the AR N-terminus that are important for transcriptional activation have been identified, notably AF-1a (residues 154–167) and AF-1b (residues 295–459), which are necessary for full transcriptional activation mediated by the receptor (3).

UXT was previously identified as an AR N-terminal coactivator and is established to interact predominantly with the AR153–336 (containing AF-1a and a part of AF-1b), which is localized to the nucleus and increases AR transcriptional activity when overexpressed in cultured mammalian cells (3). Endogenous UXT interacts with AR in nuclear extracts from lymph node carcinoma of the prostate (LNCaP) cells in a ligand-independent manner (3). Multiple studies have demonstrated that native UXT was a part of multiprotein complex which includes AR functioning in transcriptional regulation (3,4). native UXT is part of a multiprotein complex that includes proteins functioning in transcriptional regulation (3,4). Certain components of the UXT complex have been identified by mass spectrometry analysis, and UXT has been observed to associate with proteins, including RBP5, TIP48, TIP49 and other unidentified proteins (25).

ARIP3 (PIASxα) was originally identified as a testis-specific AR coregulator using yeast two-hybrid analysis with AR DBD as a bait (11). ARIP3 residues 443–548 have been suggested to be critical for the AR-ARIP3 interaction to occur, and the majority of the C-terminal region of ARIP3 containing the AR interaction domain (AR ID, residues 443–548) is unique to ARIP3 (11). The AR LBD alone was not observed to associate with the ARIP3 interaction domain (ARIP3 ID) and the N-terminal half of the AR encompassing AF-1 (residues 5–538) failed to recognize either ARIP3 ID or full-length ARIP3, indicating that AR interacts with ARIP3 ID primarily through the DBD (11). To determine whether the ARIP3 mutants maintained their ability to interact with AR, AR and FLAG-tagged ARIP3 or ARIP3 mutants were ectopically expressed in COS-1 cells (11). The results demonstrated that full-length ARIP3 and certain ARIP3 mutants (Δ1–102, Δ467–547, L23A, L304A, C385S and C388S) displayed interactions with AR that were not significantly different from each other, whereas the interaction of ARIP3 Δ347–418 with AR was markedly weaker (11). In view of the observation that the region 467–547 is sufficient for the interaction of ARIP3 with the zinc finger region of AR in yeast, it is suggested that ARIP3 interacts with AR via multiple domains (11). ARIP3 interacts with AR *in vitro* in addition to in intact mammalian cells, and is capable of modulating AR-dependent transcriptional activity. The biphasic responses suggest that ARIP3 belongs to a multisubunit coactivator complex, and its overexpression has been observed to lead to the repression of transcription when other limiting components are titrated out of the complex (11).

In the current study, UXT was demonstrated to interact with PIAS2. The yeast two-hybrid bait contained residues 9–401 of ARIP3 and the fragment was similar to the ARIP3 mutant Δ467–547, thus, the ARIP3 bait was able to bind to the AR DBD. UXT was demonstrated to be able to directly interact with the AR NTB. Combined with other coregulators, PIAS2, AR and UTX formed a multiprotein complex and further coregulated the transcriptional activity of AR. Further investigation into the functional regulatory network of the coregulators is required.

## Abbreviations

UXT: ubiquitously expressed transcript
AR: androgen receptor
STAT: signal transducer and activator of transcription
PIAS: protein inhibitor of activated STATs
DMEM: Dulbecco’s modified Eagle’s medium
SD: synthetic dropout medium
SD/-LTHA: synthetic complete medium lacking leucine, tryptophan, histidine and adenine
GFP: green fluorescent protein
HEK 293T: human embryonic kidney cell line expressing SV40 T-antigen
HeLa: human cervical carcinoma cell line
SARM: sterile α and HEAT/armadillo motif protein
Co-IP: co-immunoprecipitation
